# Effect of Bt toxin Cry1Ab on two freshwater caddisfly shredders – an attempt to establish dose-effect relationships through food-spiking

**DOI:** 10.1038/s41598-020-62055-2

**Published:** 2020-03-24

**Authors:** Antonia Pott, Mirco Bundschuh, Rebecca Bundschuh, Mathias Otto, Ralf Schulz

**Affiliations:** 1 iES Landau, Institute for Environmental Sciences, University of Koblenz-Landau, Fortstrasse 7, 76829 Landau, Germany; 2 Federal Agency for Nature Conservation (BfN), Konstantinstrasse 110, 53179 Bonn, Germany; 3 Department of Aquatic Sciences and Assessment, Swedish University of Agricultural Sciences, P.O. Box 7050, 75007 Uppsala, Sweden

**Keywords:** Environmental sciences, Limnology

## Abstract

Genetically modified organisms (GMOs), which produce *Bacillus thuringiensis* (Bt) toxins, are widely used in agriculture in some parts of the world. Despite this, ecotoxicological methods, tailored to GMOs, are lacking to assess effects on aquatic environments. With the objective to investigate a food-related exposure pathway for aquatic shredders, we used a new food-spiking method while caddisfly larvae (*Chaetopteryx* spec., *Sericostoma* spec.) served as test species. Pure Cry1Ab toxins were spiked on black alder leaf discs and subsequently used in a feeding experiment. The toxin did not influence larval mortality compared to the control. The results, however, showed significant effects on larval lipid content (*Chaetopteryx* spec.) and development (*Sericostoma* spec.) at concentrations of 17.2 and 132.4 ng Cry1Ab/mg leaf, respectively. These changes are indicative for impacts on the fitness of the specimen and thus relevant in a risk assessment context. Ultimately, the food-spiking method allowed applying different Bt toxin concentrations leading to the establishment of dose-response relationships for various response variables. The use of long test durations and sublethal endpoints (consumption, lipid content, growth, larval instars) is, moreover, advisable when testing GMO effects.

## Introduction

Genetically modified organisms (GMOs) have been cultivated for 20 years. An often applied genetic modification in agriculturally used crops is the expression of a synthetic *Bacillus thuringiensis* (Bt) toxin to achieve insect protection^1^. Bt crops integrate crystal proteins (Cry) or vegetative insecticidal proteins (Vip) in the plant tissues^2^. Target insects, which feed on Bt plants ingest the toxin, that is activated in the alkaline digestive tract and binds to the midgut epithelial membrane, which leads to pore formation and the death of target pest^2,3^. GMOs are regulated in many countries and are subject to an environmental risk assessment (ERA) including the assessment of effects from the GM-crop cultivation on non-target organisms^4^. In this respect ERA strongly focused on terrestrial effects. Only with the publication of Rosi-Marshall *et al*.^5^, which showed adverse effects from GM-maize on detritus feeding caddisflies and quantified the entry of GM maize material into the aquatic environment, also freshwater ecosystems received more attention.

Consequently, only a small number of studies dealt with the effects of GM-crops on aquatic non-target organisms (NTO)^6^. Studies with daphnids, the most frequently assessed genus, for instance, suggested a potential hazard of Bt toxin^7–9^. Studies that address the impact of GM-crop residues on detritus feeding caddisfly larvae (Trichoptera), which are taxonomically closely related to Lepidoptera targeted by some Cry toxins such as Cry1Ab, showed high variability in their responses^5,10,11^. This inconclusive picture with regards to the sensitivity of caddisfly larvae, suggests a complex interaction between toxins and the quality of plant material influencing the exposure of detritus feeding organisms and ultimately their responses.

In this context, it seems sensible to define potential exposure pathways based on which a testing strategy can be developed^12^. Waterborne exposure is likely of relatively low relevance for detritus feeding organisms as those organisms ingest plant material potentially containing Bt toxins, which likely reflects the major exposure pathway. Hence, the administration of those toxins as part of the food of these shredders^13^ might be a truly worst case scenario. This requirement comes however with a substantial challenge, that is both the Bt toxin itself and its concentration are characteristic for a given GM event. These circumstances make the establishment of a dose response relationship based on the Bt concentration in the plant material hardly possible. A potential solution may be the artificial spiking of plant material with increasing Bt-toxin concentrations, while this spiked plant material serves as food for caddisfly larvae with the aim to derive a dose response relationship. At the same time this strategy allows to replace the crop plant material by a substrate that has a higher nutritious quality for shredders. Thereby, the wellbeing of the organisms during testing is improved, specifically in situations in which responses over long exposure durations are targeted.

Against this background, the present study aimed to investigate the feasibility of this testing strategy using Cry1Ab as a model Bt-toxin at increasing concentrations on black alder (*Alnus glutinosa*) leaf discs. As earlier studies suggested caddisfly larvae susceptible to Cry1Ab, the trichopteran species *Sericostoma* spec. and *Chaetopteryx* spec. were employed as model species (supported by own yet unpublished data). Their selection is motivated by a wide distribution throughout Europe^14–19^ and their contribution to leaf litter decomposition^20,21^. Mortality, consumption, growth (case width), and larval instars (head capsule width) were recorded as response variables over a study duration of up to 12 weeks. In addition, the lipid content in caddisfly larvae was measured, as it is an important energy resource for insects during starvation and metamorphosis^22^.

## Material and Methods

### Preparation of leaf discs

Black alder (*Alnus glutinosa*) leaves were collected in 2014 from trees near Landau, Germany (49°11′N; 8°05′E). After collection the leaves were stored in a freezer at −18 °C. Some of the leaves were placed in a stream for 2 weeks to establish a natural microbial community^23^. Those leaves were later used for the conditioning (=colonization by heterotrophic microorganisms) of the leaf material used as food during the experiments. For the conditioning, leaf discs with 2 cm in diameter were cut from frozen leaves with a cork borer avoiding the main vein. These leaf discs were conditioned with leaves preconditioned in a natural stream for 10 days in medium^24^. The medium consisted of 100 mg CaCl_2_ × 2H_2_O, 10 mg MgSO_4_ × 7H_2_O, 500 mg morpholino propane sulfonic acid (MOPS), 100 mg KNO_3_ and 5.5 mg K_2_HPO_4_ per litre. By using NaOH the pH was adjusted to 7.00 ± 0.05. Afterwards the leaf discs were dried at 60 °C for 24 h. The discs were weighted and six to eight leave discs were combined per replicate. The number of leaf discs per replicate was increased with study duration, due to the enhanced food requirements of the caddisfly larvae with their growth.

### Food-spiking method

A trypsin activated Cry1Ab protein (Marianne Carey, Case Western Reserve University, Cleveland, USA) was dissolved in a buffer. The buffer contained 0.55 g CAPS-buffer and 125 µl Tween-20 (both Carl Roth, Karlsruhe, Germany) per 250 ml. The pH was adjusted to 10.5 ± 0.05 using NaOH. To dissolve the Cry1Ab protein, buffer was added in small steps to the protein, while each of these steps was followed by ultrasonication to increase dissolution. The resulting solution (538.0 ng/µl or 539.6 ng/µl, depending on the batch) was used as stock solution which was further diluted using buffer.

Before spiking, the leave discs were rehydrated in distilled water for 24 h, to prevent the discs from floating on the water surface and thus to guarantee their accessibility for the test organisms. Afterwards, the water was decanted and the leaves were gently dried with paper tissue and spiked with Cry1Ab to achieve a nominal concentration of 0, 1, 10, 100 and 1000 ng/mg leaf disc. The spiked leaf discs were let air dried for an hour and stored at −18 °C until further use.

### Test organisms

Larvae of *Sericostoma* spec. and *Chaetopteryx* spec were collected one week before the start of the respective experiment in spring 2015 from a near natural stream (49°5′N; 7°37′E) in Rhineland-Palatine, Germany. The larvae were kept in aerated stream water collected from the source stream at 16 °C on a 12:12 light:dark cycle and were fed naturally colonized black alder leaves.

### Caddisfly test

For each treatment 10 replicates were set up using 500 ml crystallizing dishes containing five randomly selected caddisfly larvae. Each dish was filled with 300 ml SAM-5S medium^25^ and 100 g natural loamy sand – the latter serving as a building material for the cases of the test organisms. SAM-5S medium contains 147 mg CaCl_2_ × 2H_2_O, 85.5 mg NaHCO_3_, 61.5 mg MgSO_4_ × 7H_2_O, 3.8 KCL and 1.03 mg NaBr per litre^25^. The sediment was collected at the same site as the larvae and was muffled for four hours at 450 °C to eliminate sediment organic material which may have served as alternative food source for the caddisfly larvae interfering with the response variables assessed. The caddisfly larvae were fed spiked leaf discs of the respective treatment over 6 (*Sericostoma* spec.) and 12 (*Chaetopteryx* spec.) weeks. In the experiment with *Sericostoma* spec. the mortality in the control after 6 weeks was already relatively high, so that we decided to terminate it. The feeding test lasted from 23. April to 16. July 2015 (*Chaetopteryx* spec.) and from 27. May to 8. July 2015 (*Sericostoma* spec.), while the environmental conditions were kept at 16 °C and on a 12:12 light:dark cycle. Every week the medium and the leaf discs were renewed and each replicate was checked for mortality. The remaining leaf discs were dried at 60 °C and weighted to the nearest 0.01 mg to determine the consumption on a weekly basis. Three additional crystallising dishes with spiked leaf discs but without caddisflies were used in order to correct the weekly consumption for microbial leaf mass loss.

To estimate the growth and the instar of the caddisfly larvae, case width and head capsule width were measured from digital images taken at the start and termination of the experiment. The measurements were realised using Axiovision (Zeiss, Jena, Germany). The larval instars were determined by the head capsule width according to Wagner^15,26^. At the start and after termination of the experiment, 15 larvae of each treatment were removed from their cases and were frozen in liquid nitrogen. Afterwards, these larvae were freeze-dried and weighted to the nearest 0.01 mg. They were kept in a −18 °C freezer until further analysis.

The lipid contents of these larvae were measured at the beginning and termination of the experiment using the vanillin-phosphor method as proposed by van Handel^27^ and modified by Zubrod *et al*.^28^. Briefly, larvae were placed for 72 h in a 1:1 chloroform:methanol solution. They were grinded with mortars and centrifuged and the supernatant were used for the lipid analysis. In a water bath the solvents were vaporised at 95 °C. Afterwards 200 µL sulphuric acids (95%) were added and the samples placed in a water bath for 10 min. The samples were let to cool down to room temperature. Five mL of vanillin-phosphor reagent were added and extinction was measured photometrically with a microplate reader (Tecan, Männedorf, Switzerland) at a wavelength of 490 nm. A 7-point calibration curve was prepared using commercial soybean oil (Sojola, Herford, Germany).

### Degradation experiment

To investigate how the Cry1Ab concentration on the spiked leaf discs developed between food renewals, a degradation experiment was performed. Therefore, spiked leaf discs were kept under the same conditions as reported for the feeding test for seven days. After day 0, 3 and 7, subsamples were removed, leaf discs were freeze-dried, weighted to the nearest 0.01 mg and stored in a −18 °C freezer until Cry1Ab quantification. For each treatment and each sampling date 5 replicates were set up.

### Cry1Ab quantification

The Cry1Ab protein concentrations of the spiked leaves from the caddisfly experiments, samples from the degradation experiment and the spiked leaf powder from the European corn borer (ECB) biotest (Fig. S8) were quantified using an enzyme-linked immunosorbent assay (ELISA)^29^. All samples were freeze-dried and if needed grounded using a mixer mill. PBST buffer was added to the sample and shaken in the mixer mill. The PBST buffer contained 8 g sodium chloride, 1.15 g sodium phosphate, 0.2 g potassium phosphate, 0.2 g potassium chloride, 0.5 g Tween-20 per litre and the pH was adjusted to 7.4 ± 0.05 with HCl. The samples were centrifuged and placed on ice. The supernatant was removed and diluted with buffer. Every sample was analysed in triplicate in an antibody coated 96-well plate (Agdia, Elkhart, USA). The well plate was shaken for 1.5 h. Then, the well plate was washed three times with the PBST buffer and an enzyme conjugate was added. The well plate was shaken for 1.5 h and washed again. A substrate solution was added. The colorimetric response was measured at the wavelength of 650 nm using a Tecan microplate reader. To quantify the Cr1Ab concentration the measured absorbance was compared with a 13-point standard curve ranging from 0 to 6.8 ng Cry1Ab/ml. The measured Cry1Ab concentrations were normalised to the sample weight. The ELISA analyses of the spiked leaves in the caddisfly feeding test revealed Cry1Ab concentrations of 0.09, 1.2, 17.2 and 132.4 ng/mg. As a consequence of the partly high deviation between the nominal and measured concentration, the present paper refers to the measured concentration in the following.

### Statistical analysis

Mortality was assessed through a time to event analysis, i.e. a log-rank Kaplan-Meier test, using SigmaPlot version 12 (Sysstat, Erkrath, Germany). For all other endpoints R software version 3.4.3 was used. Consumption per individual was measured by dividing the consumed food through the number of living larvae. In weeks, where larvae died they were counted only half assuming those individuals have been active for at least half of the week in which they died. Consumption was calculated as mg consumed leaf per larvae and week. Microbial decomposition (Md) and consumption (C) were calculated as shown in equation (1) and (2).

1 $$Md=1-\frac{({L}_{b}-{L}_{a})}{{L}_{b}}$$

2 $$C=\frac{({L}_{b}\ast Md-{L}_{a})}{S}$$

L_b_ and L_a_ is the leaf weight before and after feeding and S are the surviving larvae. Consumption was analyzed using two-way repeated measures ANOVA with experimental duration and treatment as independent factors. Mean consumption (MC) was expressed as the mean of mg consumed leaf per larvae averaged over the whole study duration. Mean consumption of all weeks, case width gain and the larval lipid content were assessed for significant differences by a one-way independent ANOVA with treatment as an independent factor followed by a Dunnett Post-hoc test. Effect sizes are expressed as a percent deviation between the control and the respective treatment. The larval instars were assessed by using a Chi^2^-Test with adjusted alpha levels (Bonferroni correction). The significance level was set at *p* < 0.05. For the degradation experiment means are plotted with a regression line and a 95% confidence interval.

## Results

### Mortality

**Figure 1:**
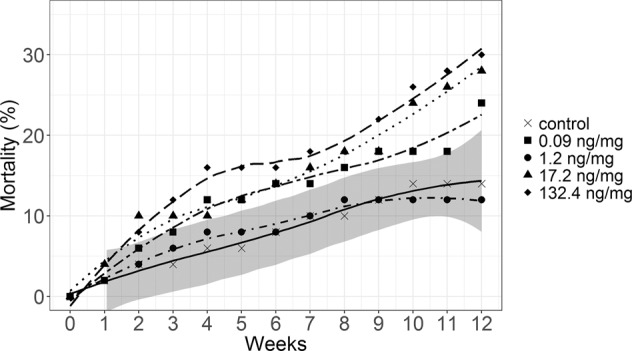
*Chaetopteryx* spec. mortality (%) over the entire study duration of 12 weeks of feeding test. Shown are means (n = 10) and regression lines. Shaded area depicts the 95% confidence band of the controls’ mean.

The mortality of *Chaetopteryx* spec. larvae increased in all treatments with study duration (Fig. 1, Table S1). The results showed, however, no statistically significant difference (*p* > 0.05) among treatments. A 2-fold higher mortality was, nonetheless, reported at the highest concentration (30 ± 25.4%) relative to the control (14 ± 19%) suggesting a concentration-dependent response. In *Sericostoma* spec. no significant difference regarding mortality was reported (Fig. S1).

### Consumption

**Figure 2:**
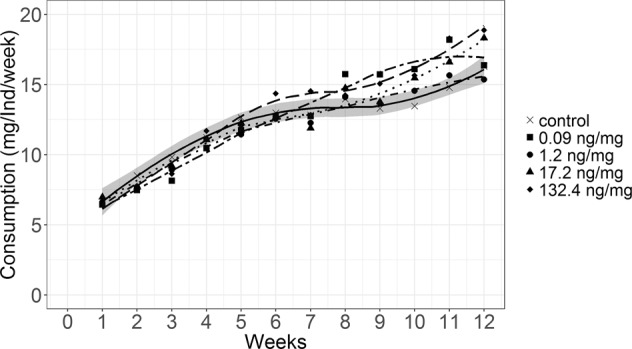
*Chaetopteryx* spec.’s leaf consumption (mg dry weight/individual/week) over the entire study duration of 12 weeks of feeding test. Shown are means (n = 10) and regression lines. Shaded area depicts 95% confidence band of the control regression.

The consumption of leaf material by *Chaetopteryx* spec. larvae increased over the study duration independent of the treatment (Fig. 2, Table S2). In the control the consumption rose from 6.7 ± 1.3 mg/ind/week to 16.3 ± 2.4 mg/ind/week at the end of the experiment. In contrast to *Chaetopteryx* spec., the consumption of *Sericostoma* spec. remained approximately the same until test termination (Fig. S2). In the control the consumption was 5.9 ± 1.6 mg/ind/week at the beginning and 6.3 ± 5.0 mg/ind/week at termination. We found no significant difference (p > 0.05) in consumption among treatments, neither for *Chaetopteryx* spec. nor *Sericostoma* spec.

By calculating the mean consumption of all weeks, we investigated, if the caddisflies changed their feeding rate which could be the result of damage in the digestive tract (Fig. S3). The mean consumption of all weeks of *Chaetopteryx* spec. and *Sericostoma* spec. larvae showed no significant difference between treatments and control (effect sizes between 1.8% and 7.5% for *Chaetopteryx* spec. and between 0.7% and 13.1% for *Sericostoma* spec.). The results revealed no dose-response relationship.

### Lipid content and case width

**Figure 3:**
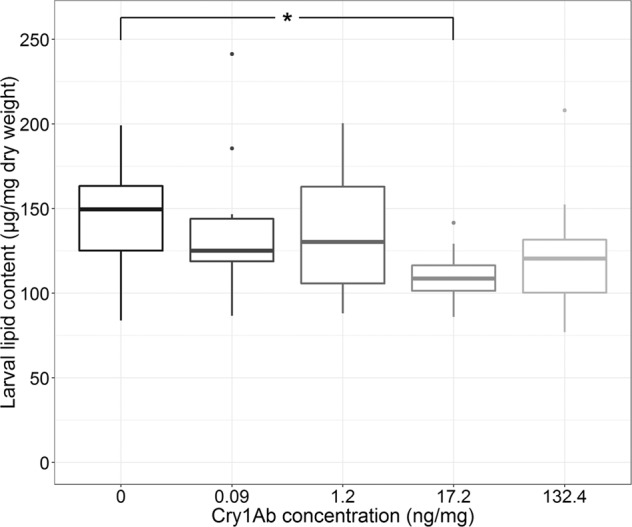
*Chaetopteryx* spec. larval lipid content after 12 weeks of feeding with Cry1Ab spiked leaf discs. Thick lines in the boxplots show medians (n = 15), lower and upper quartile are covered by the upper and lower end of the box. Effect size: 23.5%. *Shows significant difference to control (p < 0.05).

The lipid content of *Chaetopteryx* spec. was 244 ± 60 µg/mg at the beginning of the experiment. At the termination of the experiment, the lipid content decreased with increasing Cry1Ab concentration, while only for the 17.2 ng Cry1Ab/mg treatment a significant reduction relative to the control was observed (effect size of approximately 25%; Fig. 3, Table S3). This observation was not confirmed for *Sericostoma* spec (Fig. S4).

The case width of the *Chaetopteryx* spec. larvae increased in all treatments over the experimental duration. The results showed a significant lower case width gain (p < 0.05) in the highest Cry1Ab treatment relative to the control (Fig. S5). Moreover, at the termination of the experiment, the caddis width differed less than 5% among treatments. Similarly, there was no effect on *Sericostoma* spec. detectable (Fig. S6).

### Larval instars

**Figure 4:**
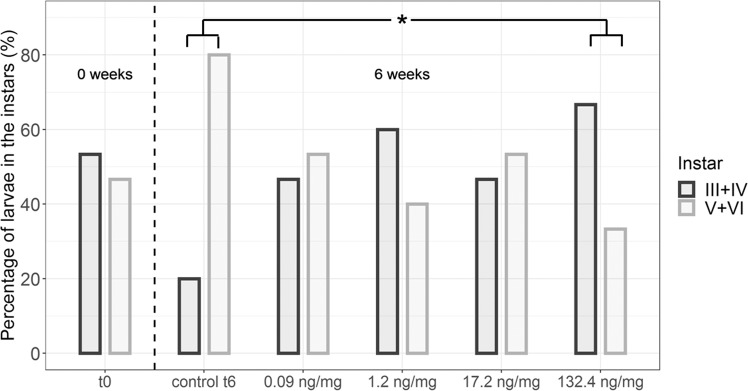
*Sericostoma* spec. larval stage at the start (t0) and the termination (t6) of the experiment in the control (control t6) and the various treatments, respectively (n = 15). The dashed line separates the data of the larval instars at the beginning (0 weeks) and at the end (6 weeks) of the experiment. Larval stage III was found in the 17.2 ng/mg treatment. Larval stage VI was found in the 0.09 and 1.2 ng/mg treatment. *Shows significant difference to control (p < 0.05).

At the beginning of the caddisfly experiment, the *Sericostoma* spec. larvae in the control were almost equally distributed among instars ≤IV and ≥V (Fig. 4). The Chi^2^-Test showed a significant difference (p > 0.05) in larval instars after 6 weeks, i.e. larval development was delayed, between the highest concentration and the control (Table S3). This pattern in larval instars could not be confirmed by the experiments with *Chaetopteryx* spec (Fig. S7).

### Degradation experiment

**Figure 5:**
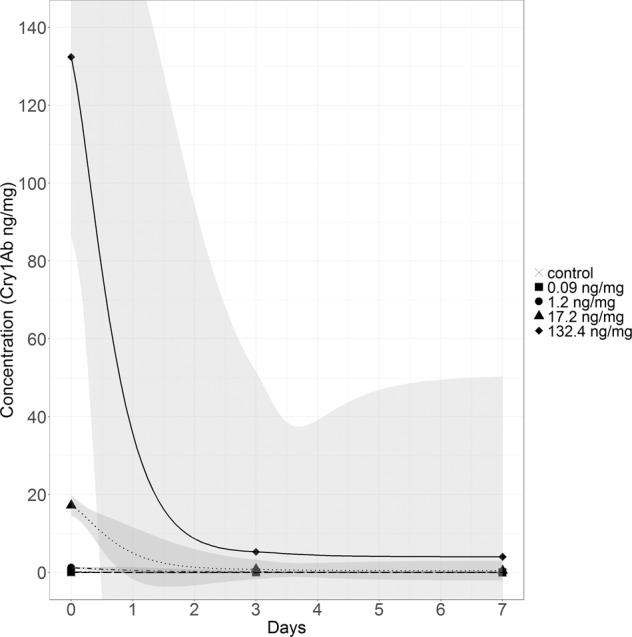
Cry1Ab concentration on spiked leaf discs during 7 days stored in medium. Shown are means (n = 5), regression line and 95% confidence interval. No Cry1Ab toxin was measured in the control.

The degradation experiment showed a rapid decline of the Cry1Ab concentration on leaves during one week (Fig. 5). At the highest concentration (132.4 ng/mg), for instance, a decrease to 4.01 ng/mg was observed after 7 days. No Cry1Ab toxin was measured in the control.

## Discussion

### Bt effects on caddisflies

Despite their close taxonomic relationship to Lepidoptera, which are targeted by a variety of Bt toxins expressed in transgenic crops, only few studies examined the hazard of Bt toxins on caddisflies^6^. Our results add to that knowledge and suggest that at least some groups of caddisfly larvae may indeed be sensitive to the Cry1Ab toxin when exposed through their food over an extended period of time. Lethal effects of Bt toxins on caddisflies have been so far only shown in Rosi-Marshall *et al*.^5^ for the scraping caddisfly *Helicopsyche borealis* when fed pollen from a Cry1Ab maize event. Most of the other studys^10,11^ demonstrated sublethal effects on caddisfly larvae after exposure to Bt toxins. Observations of Chambers *et al*.^11^ and Rosi-Marshall *et al*.^5^ highlight a significantly lower growth of *Lepidostoma liba* larvae when fed Cry1Ab containing maize leaves compared to non-Bt maize. Divergent observations in other Trichopteran species such as *Pycnopsyche* cf. *scrabripennis* document an increase in biomass when larvae were exposed to Cry1Ab × Cry3Bb1 × Roundup Ready maize leaves compared to the exposure to either Roundup Ready maize or Cry1Ab maize^10^. In fact a uniform response of all Trichopteran species to Bt toxin, or a special class of Bt toxin, cannot be expected as Lepidoptera show a large inter-specific variation in their sensitivity^30^.

For Trichoptera, we suggest that sublethal responses can indeed be more informative for the risk assessment relative to mortality, because especially at chronic low exposure, the damage of the gut membrane caused by Bt toxins is known not to be lethal^31–34^. In our case decreasing larval lipid content and retarded insect development indicated impairments, though often not statistically significant, this may be mainly related to the rather high variability in those variables and the relatively low number of replications and thus statistical power and the quick degradation of the spiked toxin.

The trend to a lower larval lipid content in *Chaetopteryx* spec. with increasing Cry1Ab concentrations may indicate a reallocation of energy towards defence and repair mechanisms while maintaining growth^35^. The significant lower case width gain in the highest Cry1Ab concentration may be mainly driven by the significant differences in the case width at the start. A reallocation of energy may, however, have negative implications in the ability of the organism to withstand future challenges such as metamorphoses and their ability to reproduce in the terrestrial life stage^35^. In contrast to *Chaetopteryx* spec., the significant difference in the larval stages of *Sericostoma* spec. among treatments at the termination of the experiment indicates – although variability could not be defined – together with non-substantial changes in their lipid content that ensuring sufficient energy reserves for future investments (e.g. number of offspring) is favoured over the speed of development. The slower development of the larval instars in some of the Cry1Ab treatments, likely postpones emergence which might temporarily disconnect the provisioning of prey from aquatic recourses to terrestrial food webs potentially inducing bottom-up or top-down directed effects^36^. However, it has to be mentioned that in our study the control and the highest concentration obviously contained already different *Sericostoma* spec. instars at the beginning of the experiment. A delay of the development of larval instars by Cry1Ab toxin was also reported for terrestrial lepidopteran larvae^30,37^ and for caddisfly larvae when exposed to pesticides^38–40^. Consequently, alterations in the physiology (i.e., lipid reserves) and larval instars of insects as a consequence of exposure to stress in general, and Cry1Ab in particular, seems a common response suggesting that the subtle effects observed in the present study warrant further research.

The Cry1Ab concentrations in leaves of MON810 and a not specified event are on average approx. 5 ng/mg^41^ and 10.2 ng/mg (values between 7.3 and 15.1 ng/mg)^42^, respectively. Maize debris collected in the riparian zone outside the water show lower Cry1Ab concentrations (mean 0.20 ± 0.34 ng/mg)^43^. Cry1Ab leaches from maize detritus into the water, so concentrations in maize leaves collected in a stream can be expected to be lower than in the riparian zones. Cry1Ab in maize detritus in streams showed concentrations of Cry1Ab in average of approx. 1 ng/mg, and 0.095 ± 0.073 ng/mg^41,43^. For the lipid content we found significant effects at a concentration of 17.2 ng/mg, which are similar to Cry1Ab concentrations found in fresh maize leaves. At a concentration of 132.4 ng/mg the larval instars showed significant sublethal effects and, thus, approximately tenfold above Cry1Ab concentrations in fresh Bt-maize leaves. Our sublethal effects were found at concentrations ten- to thousand folds above the Cry1Ab concentrations in maize detritus from the stream or the riparian zone. The results indicate that significant effects may occur if fresh Bt-maize leaves are present. The risk seems lower if looking at Bt-maize material from the stream or the riparian zone. However, SmartStax, a stacked GM maize expressing six Cry toxins shows clearly higher concentrations in leaves (total Cry toxin concentration 1518 ng/mg)^44^. Moreover, risk assessment should cover possible uncertainties arising from species differences and the extrapolation from acute to chronic tests. For this purpose, assessment factors (e.g. 10, 100 or 1000) are used especially for the determination of Predicted No Effect Concentrations (PNEC). Furthermore, due to the limited data, this assessment is subject to uncertainty. This illustrates the need for further verification studies, especially because of the specific mode of action of the toxins.

### Relevance of the food-spiking method

With the food spiking method, it was ensured that the observed effects are triggered by the Bt toxin and not by the quality of the leaf material, as the latter was prepared in a standardised manner. To highlight the strength and limitations we discuss the proposed food spiking method in the context of other published methods involving GMO and caddisflies^6^. In contrast to our study, recently published studies often did not quantify the Bt toxin concentration in the applied plant material and were restricted to one or two treatment concentrations^5,10,11^. However, different concentrations are necessary to obtain dose-response relationship and abstract related effect thresholds, which are important input data for risk assessment. The food-spiking method, as applied here, allowed establishing different concentrations that were supported via ELISA and it is thus suitable to investigate dose-response relationships. Indeed the present study allowed identifying concentrations of Cry toxins that affected the test species in a sublethal manner. Those effect concentrations can be compared to concentrations actually measured in crop plants ultimately informing risk assessment. In the following we look at further aspects that highlight the relevance of the proposed approach.

Moreover, our additional experiments involved biotests with *Ostrinia nublilalis*, a target insect pest of Cry1Ab maize. These biotests clearly demonstrated that the spiking method is suitable to expose the test organisms and thus to assess food related effects on aquatic shredders. As shredders are an important functional group in aquatic systems the method can be a valuable tool for the assessment of effects of GMO cultivation on aquatic environments^12^.

To compare the GM-plant and its isogenic control line is the basis of the assessment in many studies^7,10,45,46^. Feeding studies, particularly those involving a non-GM isogenic line as a control, may fail of providing equally nutritious food for shredders in both the treatments and the control, complicating any conclusion on potential environmental risks of the toxin. Studies measured differentially expressed proteins in a GM-maize relative to its non-transgenic isogenic line, however, the biological relevance of such changes is still unknown^47,48^. Because nutritional quality of food leaves is the same both in the control and treatments groups, our food-spiking method strongly suggests that the responses of caddisfly shredders are largely triggered by the toxin. The GM-plant material used in some recently published studies was conditioned, i.e. microbially colonized for 3, 7 or 14 days^5,10,11^. We agree that the conditioning is essential for shredding organisms as it improves the food quality. At the same time, conditioning can lead to uncontrolled degradation and leaching of the Bt toxin into the water phase^49^. In our food-spiking method leaf conditioning took place prior to spiking, thus, we excluded the risk of a Bt toxin degradation during the conditioning process. Nonetheless degradation and dilution during the feeding experiment cannot be avoided and the spiking method itself requires further verification as we detected only roughly 10% of the nominal concentration at test initiation.

Studies reported in the literature were often limited to test durations of up to 30 days. The entry of maize debris into the water starts in October after harvest, is highest in February and stops after growth of new vegetation in April^10^. Aquatic organisms are thus exposed for a long time period to low Bt toxin concentrations in the environment^41,42,50^. In order to realistically represent the conditions in the environment, we chose an exposure time of 6 and 12 weeks. We conclude that using such prolonged exposure time is more closely reflecting the actual field scenario and thus consider this as an advantage when investigating Bt toxins.

The degradation experiment showed a rapid decrease of the Bt toxin concentration on the leaf discs during one week in the water. This resembles the real exposure in a stream, which is probably also not constant. To achieve a more even Bt toxin level, the spiked leaf discs may be exchanged more often.

To conclude, we showed significant effects on some endpoints and our data suggest concentration-dependent responses. Effects were found, however, at higher concentrations and were not consistent in all species and all endpoints. We, nonetheless, showed that long test durations and the use of sublethal endpoints are reasonable and may assist assessment of risks. From our results we concluded that the food-spiking method is able to realise a food-related exposure of shredders which could inform risk assessment, but this is only a first step and additional studies would be beneficial to develop this method further.

## Supplementary information

Supplementary material.
