# Supplementary material for: Effect of Bt toxin Cry1Ab on two freshwater caddisfly shredders – an attempt to establish dose-effect relationships through food-spiking
Source: Sci Rep. 2020 Mar 24;10:5262. doi: 10.1038/s41598-020-62055-2 (PMC7093423; doi:10.1038/s41598-020-62055-2)
Supplement: Supplementary file 1 — Supplementary material. [file 41598_2020_62055_MOESM1_ESM.pdf]

Supplementary Information for:
Effect of Bt toxin Cry1Ab on two freshwater caddisfly shredders – an attempt to establish dose-effect relationships through food-spiking

Antonia Pott[1,2*], Mirco Bundschuh[1,3], Rebecca Bundschuh[1], Mathias Otto[2], Ralf Schulz[1]

[1] iES Landau, Institute for Environmental Sciences, University of Koblenz-Landau, Fortstrasse 7, 76829 Landau, Germany

[2] Federal Agency for Nature Conservation (BfN), Konstantinstrasse 110, 53179 Bonn, Germany

[3] Department of Aquatic Sciences and Assessment, Swedish University of Agricultural Sciences, P.O. Box 7050, 75007 Uppsala, Sweden

Table S1 *Chaetopteryx* spec. mortality (%) during the 12 weeks of feeding test. Shown are means (n=10) and standard deviation (SD).

Week	**Bt concentration (ng Cry1Ab/mg leaf disc DW)**									
	**control mean**	**control SD**	**0.09 mean**	**0.09 SD**	**1.2 mean**	**1.2 SD**	**17.2 mean**	**17.2 SD**	**132.4 mean**	**132.4 SD**
**1**	2.0	6.3	2.0	6.3	2.0	6.3	4.0	8.4	2.0	6.3
**2**	4.0	8.4	6.0	13.5	4.0	8.4	10.0	14.1	8.0	16.9
**3**	4.0	8.4	8.0	14.0	6.0	9.7	10.0	14.1	12.0	21.5
**4**	6.0	9.7	12.0	16.9	8.0	10.3	10.0	14.1	16.0	20.7
**5**	6.0	9.7	12.0	16.9	8.0	10.3	12.0	14.0	16.0	20.7
**6**	8.0	10.3	14.0	21.2	8.0	10.3	14.0	13.5	16.0	20.7
**7**	10.0	10.5	14.0	21.2	10.0	14.1	16.0	15.8	18.0	22.0
**8**	10.0	10.5	16.0	20.7	12.0	14.0	18.0	17.5	18.0	22.0
**9**	12.0	14.0	18.0	19.9	12.0	14.0	18.0	17.5	22.0	22.0
**10**	14.0	19.0	18.0	19.9	12.0	14.0	24.0	20.7	26.0	25.0
**11**	14.0	19.0	18.0	19.9	12.0	14.0	26.0	19.0	28.0	27.0
**12**	14.0	19.0	24.0	22.7	12.0	14.0	28.0	16.9	30.0	25.4

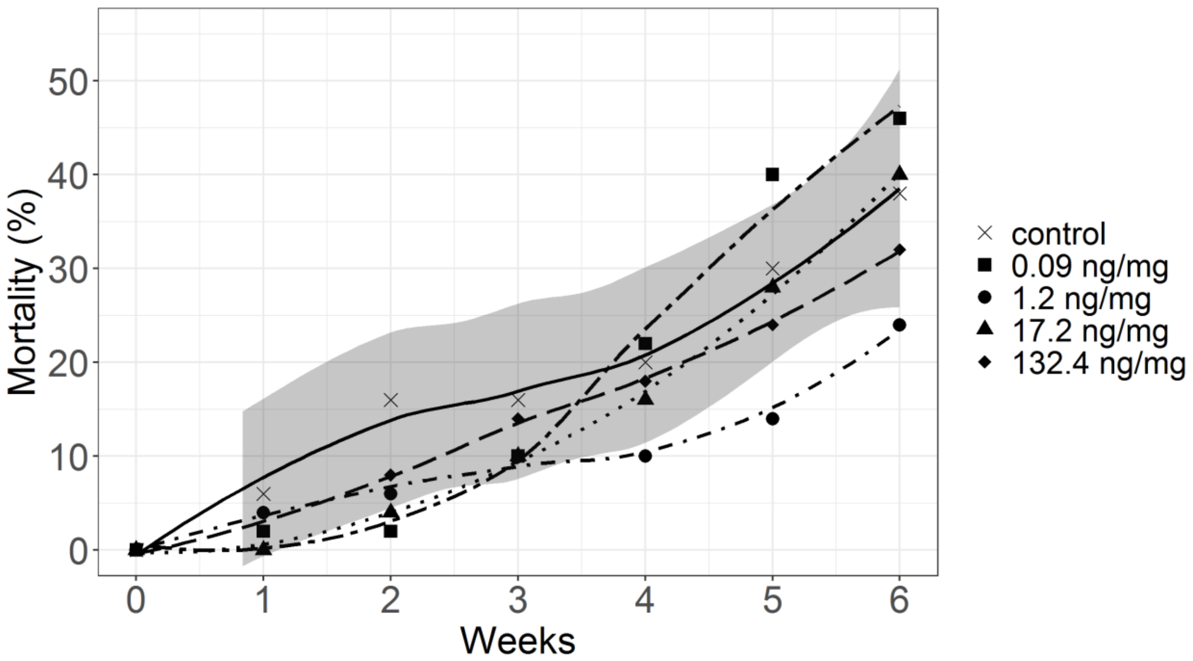

Figure S1 *Sericostoma* spec. mortality (%) over the entire study duration of 6 weeks of feeding test. Shown are means (n=10) and regression lines. Shaded area depicts the 95% confidence band of the controls' mean

Table S2 *Chaetopteryx* spec. consumption (mg leaf DW/individual/week) during the 12 weeks of feeding test. Shown are means (n=10) and standard deviation (SD).

	**Bt concentration (ng Cry1Ab/mg leaf disc DW)**									
**Week**	**control mean**	**control SD**	**0.09 mean**	**0.09 SD**	**1.2 mean**	**1.2 SD**	**17.2 mean**	**17.2 SD**	**132.4 mean**	**132.4 SD**
**1**	6.7	1.3	6.4	1.6	6.6	1.2	7.0	2.7	6.4	2.0
**2**	8.6	1.3	7.6	2.5	7.8	1.4	7.9	1.9	7.6	1.7
**3**	10.0	1.8	8.3	2.0	9.0	1.8	9.6	3.0	9.2	3.4
**4**	11.2	1.5	10.6	2.4	11.2	1.7	11.1	2.9	11.8	3.0
**5**	12.8	1.6	12.2	3.3	11.6	1.3	12.2	3.1	12.4	2.6
**6**	13.0	1.4	12.7	2.7	12.5	1.7	12.8	3.0	14.4	4.4
**7**	13.0	1.8	13.1	2.6	12.3	1.8	12.0	3.6	14.5	4.0
**8**	14.1	1.4	15.7	4.7	14.4	3.1	15.0	3.2	14.4	3.1
**9**	13.3	1.5	15.9	5.0	13.8	3.1	14.0	3.7	15.1	4.6
**10**	13.7	1.6	16.3	4.2	14.5	3.6	15.5	2.9	16.1	4.5
**11**	15.2	3.7	18.2	6.6	15.6	3.0	17.6	17.6	19.3	7.9
**12**	16.3	2.4	16.4	4.3	15.3	3.6	18.5	3.5	19.4	7.0

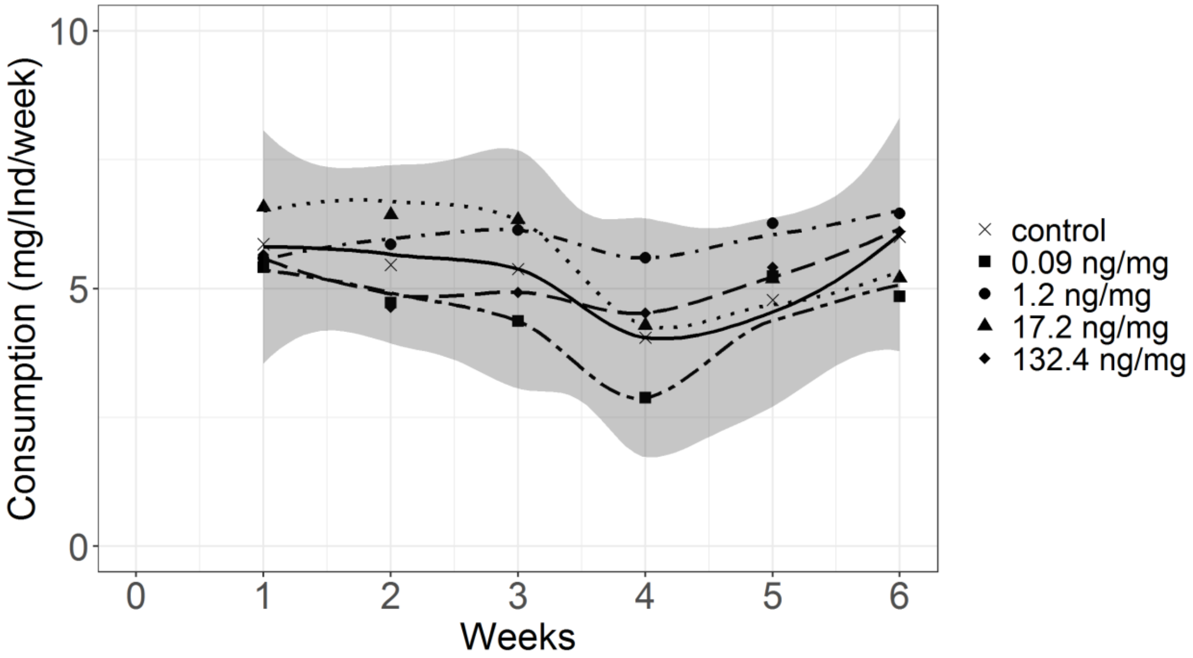

Figure S2 *Sericostoma* spec.'s leaf consumption (mg dry weight/individual/week) over the entire study duration of 6 weeks of feeding test. Shown are means (n=10) and regression lines. Shaded area depicts 95% confidence band of the control regression

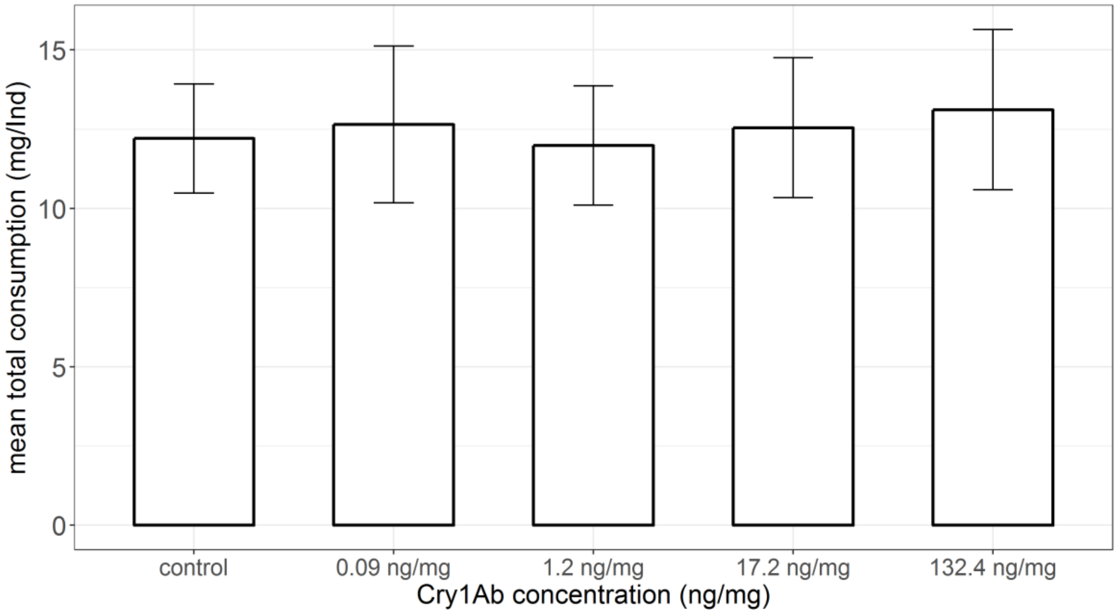

Figure S3 *Chaetopteryx* spec. mean consumption per week (mg dry weight/individual) in the feeding test. Shown are means (n=12) and standard deviation

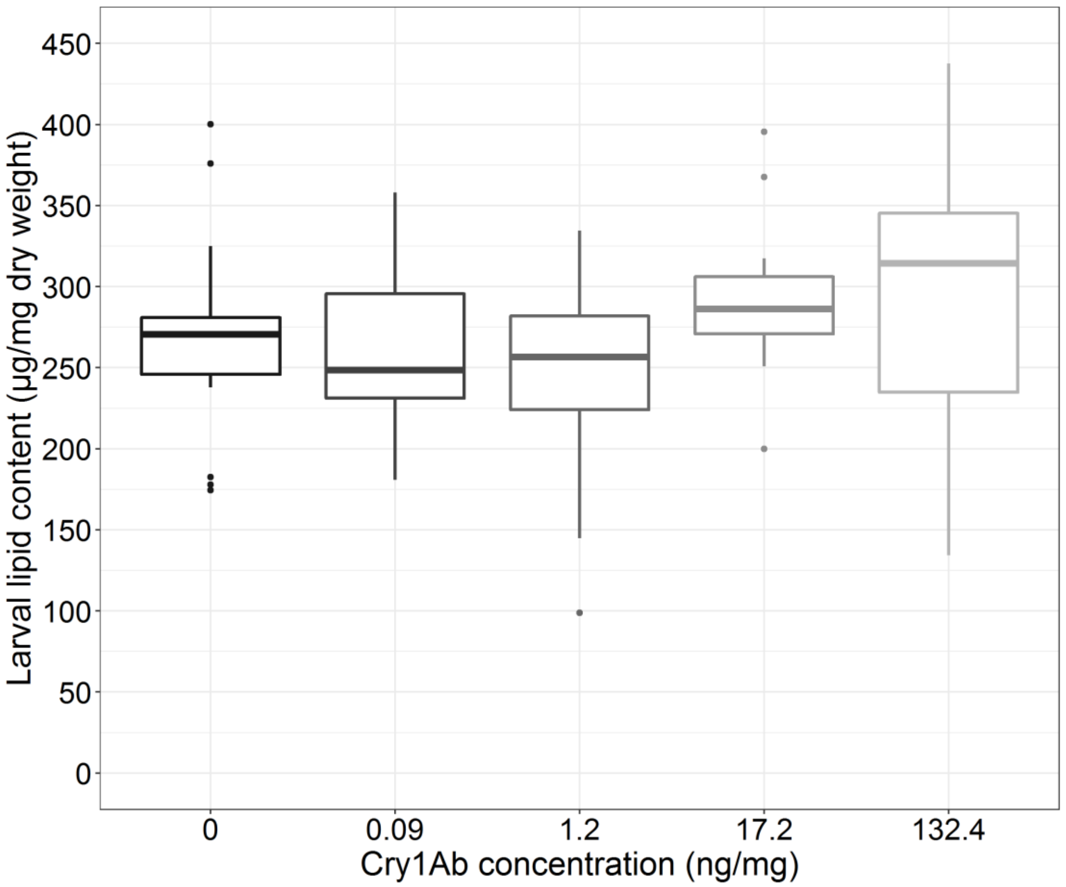

Figure S4 *Sericostoma* spec. larval lipid content after 6 weeks of feeding with Cry1Ab spiked leaf discs. Thick lines in the boxplots show medians (n=15), lower and upper quartile are covered by the upper and lower end of the box.

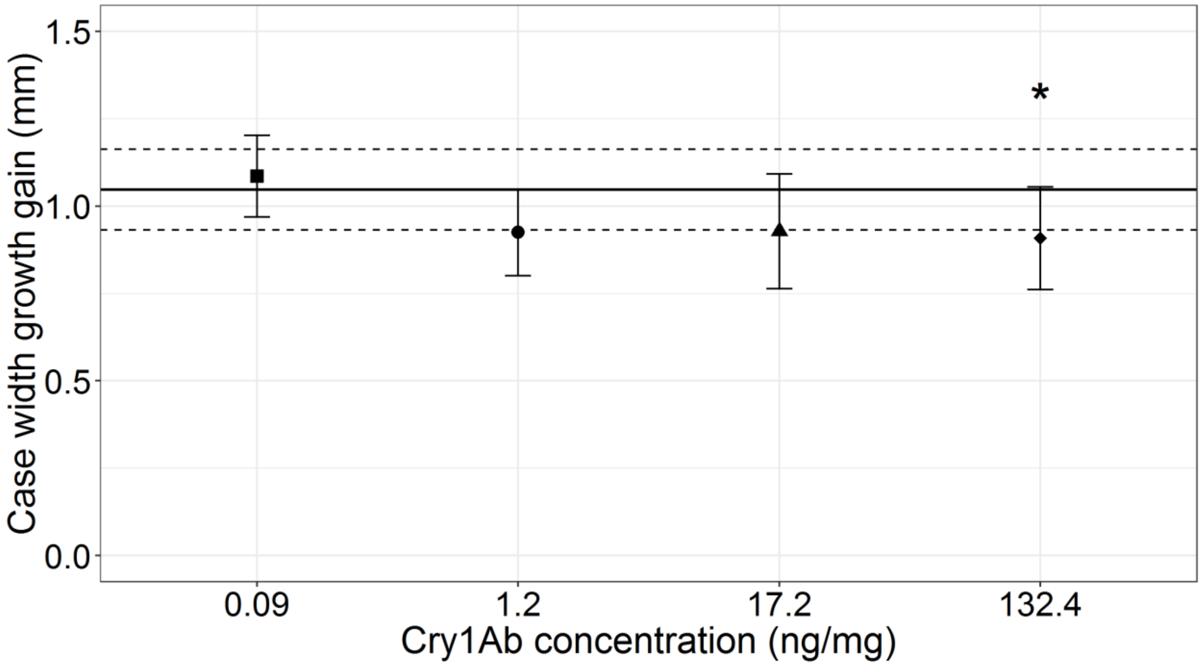

Figure S5 *Chaetopteryx* spec. growth gain of the caddis width after 12 weeks feeding with Cry1Ab spiked leave discs. Shown are medians (n=10) and 95% confidence interval. The solid and dashed lines show the median and the 95% confidence interval of the control. Effect size: 25.8%

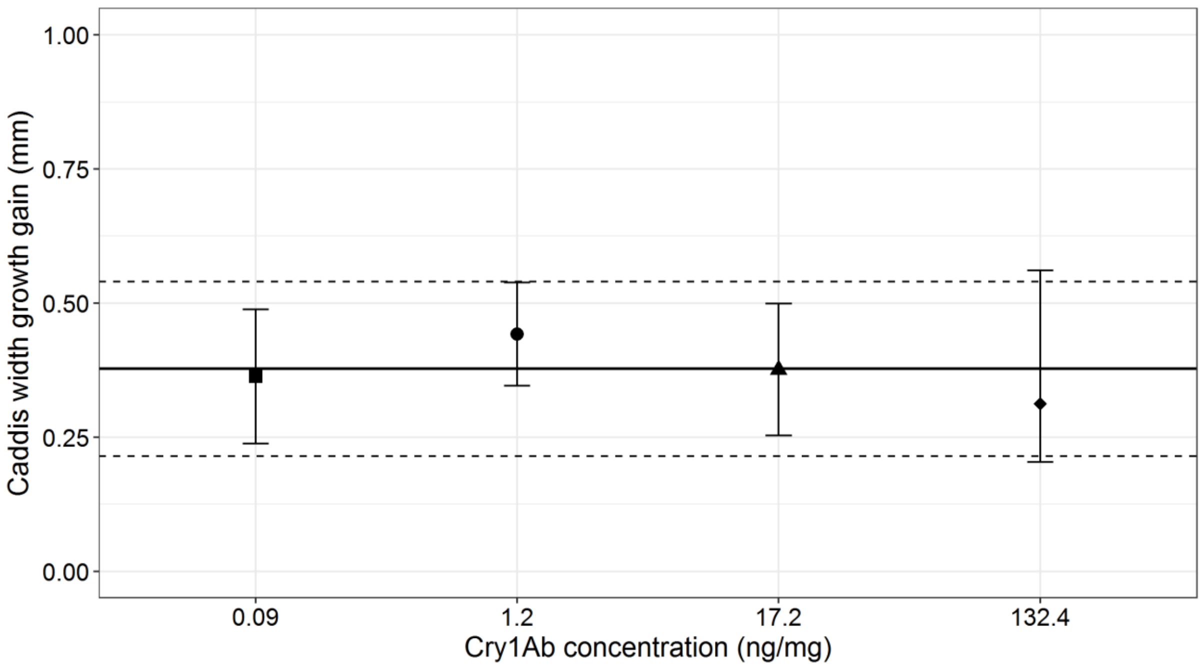

Figure S6 *Sericostoma* spec. growth gain of the caddis width after 6 weeks feeding with Cry1Ab spiked leave discs. Shown are medians (n=6-10) and 95% confidence interval. The solid and dashed lines show the median and the 95% confidence interval of the control.

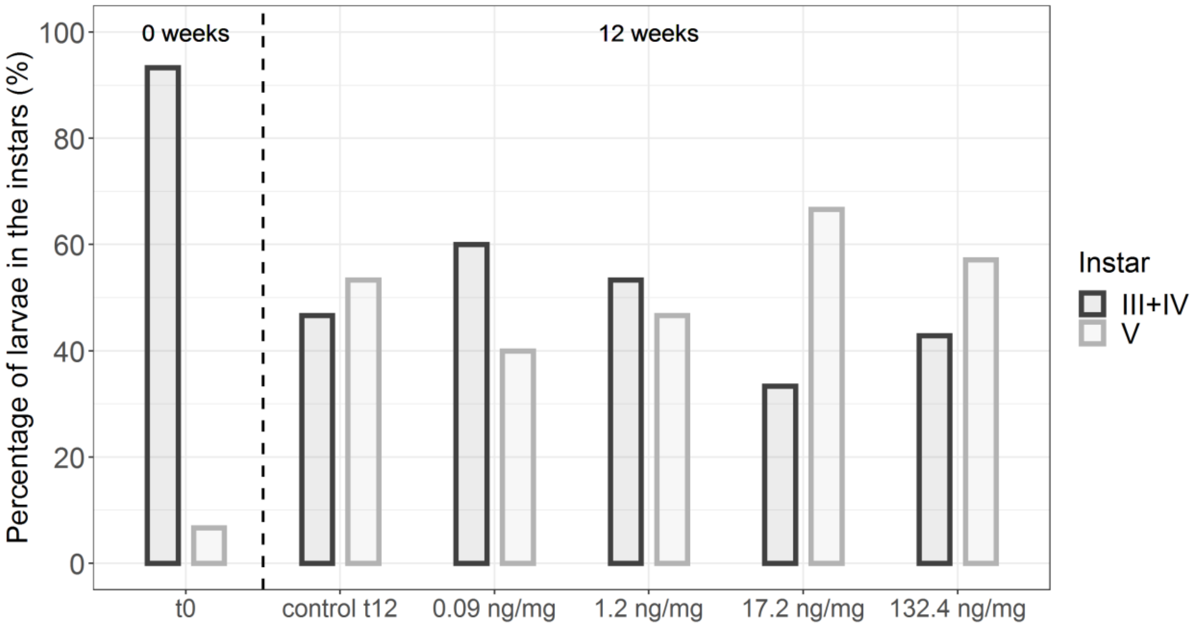

Figure S7 *Chaetopteryx* spec. larval stage at the start (t0) and the termination (t12) of the experiment in the control (control t12) and the various treatments, respectively (n=14-15). The dashed line separates the data of the larval instars at the beginning (0 weeks) and at the end (12 weeks) of the experiment. Larval stage III was found in control t12.

Table S3 Results of the statistical analysis. Shown are p-valuse and effect sizes for significant effects between Cry1Ab concentrations and the control. For the larval instars of *Sericostoma* spec. effect sizes are shown for the instars III+IV and V+VI.

Species	Endpoints	Concentration(ng/mg)	p-value	Effect size (%)
Chaetopteryx spec.	lipid content	17.2	0.0152	23.5
Chaetopteryx spec.	growth (case width)	132.4	0.0178	25.84
Sericostoma spec.	larval instars	132.4	0.0099	-233,3/ 58,3

European corn borer biotest

The European corn borer (*Ostrinia nubilalis*) is a target organism of Cry1Ab protein. In order to investigate, if the Cry1Ab protein used in the caddisfly experiment is bioactive and reaches its target site, biotests with larvae of the European corn borer (ECB) (Crambidae; *Ostrinia nubilalis*) (Annette Herz, Julius Kühn Institute, Darmstadt, Germany) were carried out. ECB eggs were reared in a climate chamber at 25±1°C, a relative humidity of 60% and a 16:8 L:D cycle. The procedure followed Conradin[1]: After hatching, the larvae were fed rearing diet ad libitum[2]. The diet contained 390 ml distilled water, 10 g agar, 25 g corn semolina, 25 g wheat germ, 25 g yeast powder, 0.9 g benzoic acid, 0.9 g nipagin and 2.25 g ascorbic acid per 0.5 l. At an age between 24 and 48 h, each larva and a small piece of moistened paper tissue were placed separately in rearing trays (C-D International, INC. Pitman, NJ, USA) to run the bioassay under conditions as detailed for rearing. To prepare the respective food, black alder leaves were dried at 60°C for 24 h and ground. Subsequently, 200 mg leaf powder was filled in vessels and Cry1Ab was added at increasing concentrations from its respective stock. The Cry1Ab spiked leaf powder was mixed with rearing diet targeting seven concentrations (0.49, 1.95, 7.81, 31.25, 125, 500, 2000 ng Cry1Ab/g). A control with diet and pure plant material and a control with diet only were also used. Subsamples from the spiked leaf powder were taken to analyze the Cry1Ab concentration and were stored in a -18 °C freezer. The bioassay duration was 7 days and the mortality was recorded every 24 h. The larvae were considered dead if after gentle touches with a brush no movement was observed. Although the spiking methods and the environmental conditions differed among experiments with ECB and the caddisfly species, we argue that the insights with regards to the biological activity of the Cry1Ab toxin applied through this method is not affected substantially.

The dose-response curve for *Ostrinia nubilalis* was prepared using a log-logistic model in the drc package[3] in R. The model that fits the data best were selected based on the model's AIC (Akaike information criterion).

The Cry1Ab concentrations on the spiked leaf powder as used during the ECB biotest uncovered measured concentrations of 0.09, 0.4, 2.6, 11.4, 58.8, 228.8 and 1087.7 ng/g. The ECB showed an increasing mortality with increasing Cry1Ab concentration and, thus, revealed a clear dose-response curve (Fig. S8). The calculated LC_{50} of 1.011 ng Cry1Ab/g is clearly lower than in another study with LC_{50} values between 100 and 2120 ng/g[4]. The mortality of the European corn borer in our study shows that the Cry1Ab toxin reached the target site in the larva. This finding could be transferred to the caddisfly test and verifies the bioactivity of the used Cry1Ab toxin. For tests with GMO a positive control often does not exist, which makes the proof of the bioactivity even more important.

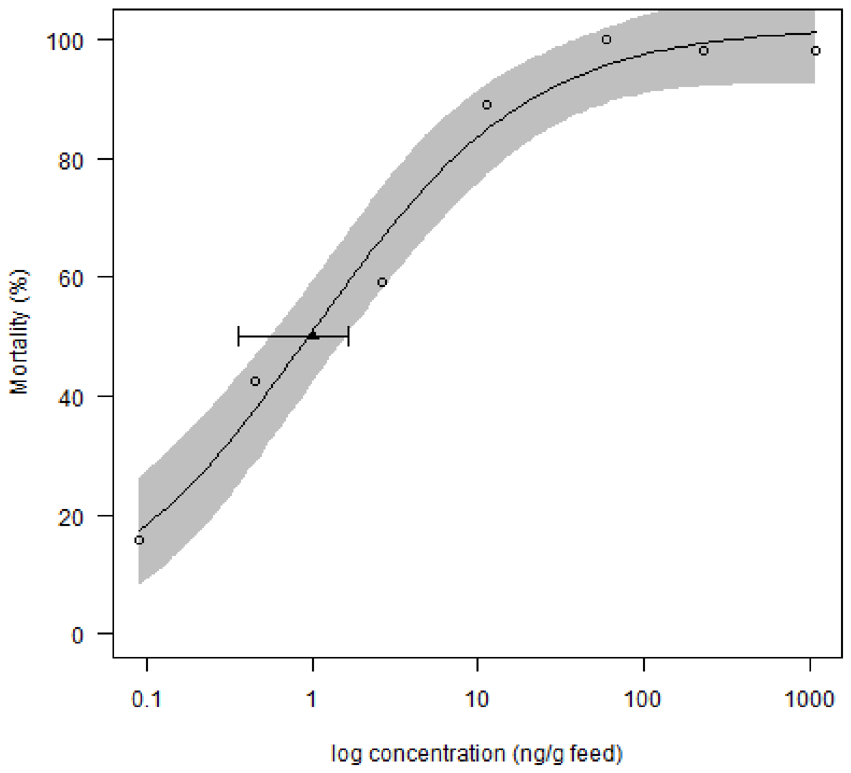

Figure S8 European corn borer mortality after 7 days of feeding with Cry1Ab spiked feed (n=3). Shown are means (n=3), dose-response curve and the 95% confidence interval. Triangle: LC_{50}=1.011 ng/g, 95% CI lower level: 0.359, upper level: 1.66. LC_{90}=28.499 ng/g, 95% CI lower level: -14,933, upper level: 71.931

References

1. Conradin, C. *Effect of antibiotics on the insecticidal activity of Bt-corn and DELFIN® toward the European corn borer Ostrinia nubilalis*. Semester thesis (2010).

2. Ivaldi-Sender, C. Simple techniques for a permanent breeding of oriental fruit moth *Grapholita molesta* Lepidoptera Torticidae on artificial diet. *Ann Zool Ecol Anim* **6,** 337–343 (1974).

3. Ritz, C., Baty, F., Streibig, J. C. & Gerhard, D. Dose-Response Analysis Using R. *PloS one* **10,** e0146021; 10.1371/journal.pone.0146021 (2015).

4. Saeglitz, C. *et al.* Monitoring the Cry1Ab susceptibility of European Corn Borer in Germany. *J Econ Entomol* **99,** 1768–1773; 10.1093/jee/99.5.1768 (2006).
